# Supplementary material for: Clarithromycin overcomes stromal cell-mediated drug resistance against proteasome inhibitors in myeloma cells via autophagy flux blockage leading to high NOXA expression
Source: PLoS One. 2023 Dec 1;18(12):e0295273. doi: 10.1371/journal.pone.0295273 (PMC10691716; doi:10.1371/journal.pone.0295273)
Supplement: S1 File — (DOCX) [file pone.0295273.s001.docx]

**S1 File. Supplementary information**

**List of S1 File.**

**Figure S1.** Establishment of a co-culture experimental system for EGFP-labeled multiple myeloma　(MM) cell lines and stromal cell lines.

**Figure S2.** Combined treatment with carfilzomib (CFZ) and clarithromycin (CAM) enhances ATF3 and NOXA.

**Supplementary Movies (S5 File).**

Confocal microscopy for monitoring the interaction between MM cells and stromal cells.


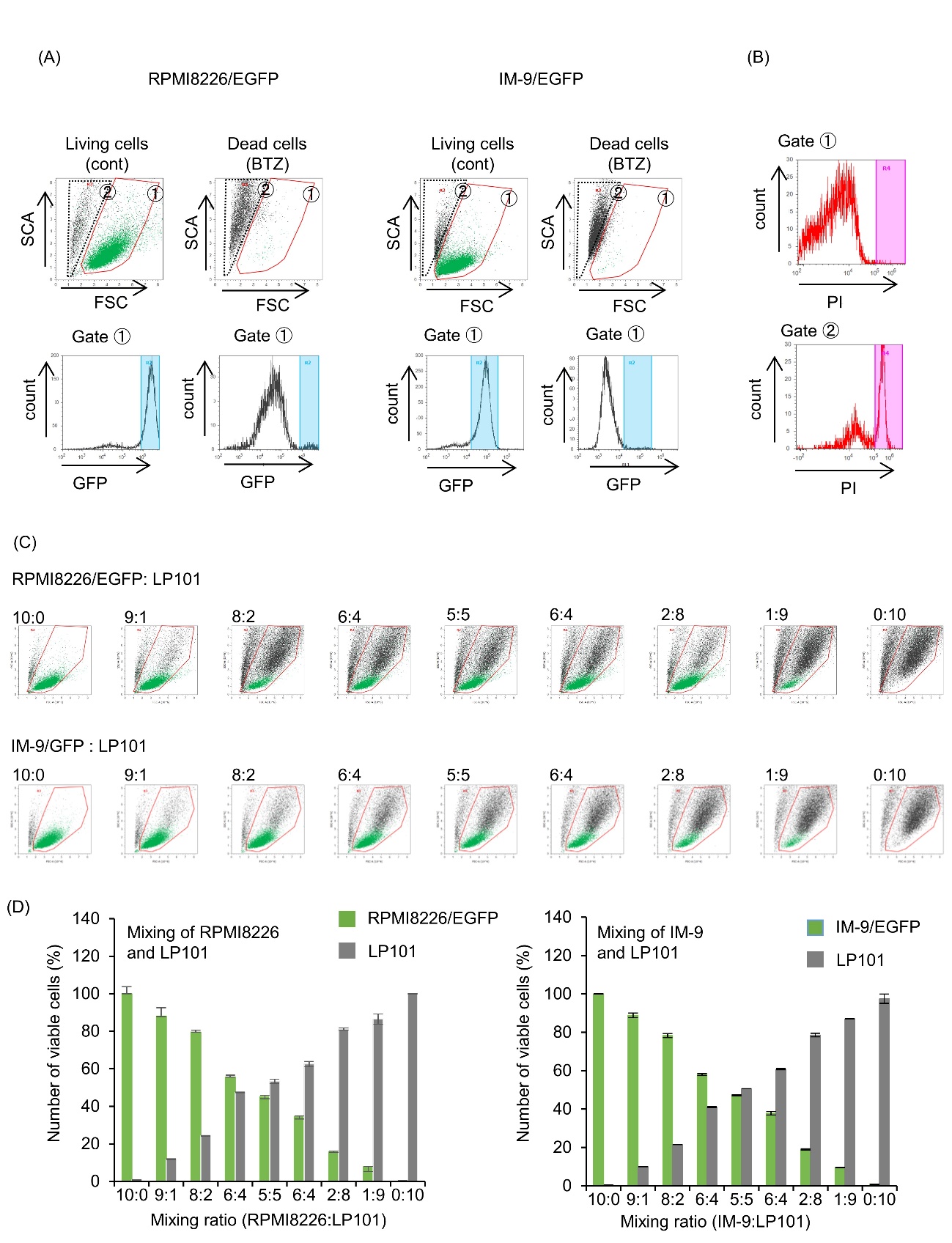


**Supplemental Fig. 1**

**Establishment of a co-culture experimental system for EGFP-labeled multiple myeloma (MM) cell lines and stromal cell lines.**

(A) RPMI8226/EGFP and IM-9/EGFP cells were cultured in the presence or absence of bortezomib (BTZ, 25 nM) for 24 h. Cells were analyzed by flow cytometry as described in the Materials and Methods. In the flow cytometric analysis, viable cells were detected as forward scatter (FSC)-positive and side scatter (SCA)-positive. The red frame is defined as viable cell gating by forward scatter (FSC)-positive and side scatter (SCA)-positive, along with PI-negative staining. The green dots inside the red frame indicate viable MM cells labeled with EGFP. The living cells were strongly positive for EGFP; however, dead cells rapidly lost EGFP fluorescence.

(B) IM-9/EGFP cells in Supplemental Fig. 1A were PI-stained and analyzed in a flow cytometer, as described in the Materials and Methods. Cells in gate ① were negative for PI staining, indicating that they were alive. In contrast, the cells in gate ② that frame out from the red box are PI positive, indicating that these are dead cells.

(C) EGFP-labeled MM cells (RPMI8226/EGFP and IM-9/EGFP) and LP101 cells were prepared at 1 × 10^6^ cells/ml. Cells were mixed at the indicated ratios and analyzed by flow cytometry, as described in the Materials and Methods. The green dots inside the red frame indicate live EGFP-labeled MM cells.

(D) Quantification of Supplemental Fig. 1C. The number of viable EGFP-positive myeloma cells and EGFP-negative LP101 cells was assessed. The number of viable cells was determined to be 100% for each cell alone. Data are presented as mean ± SD; n = 3.


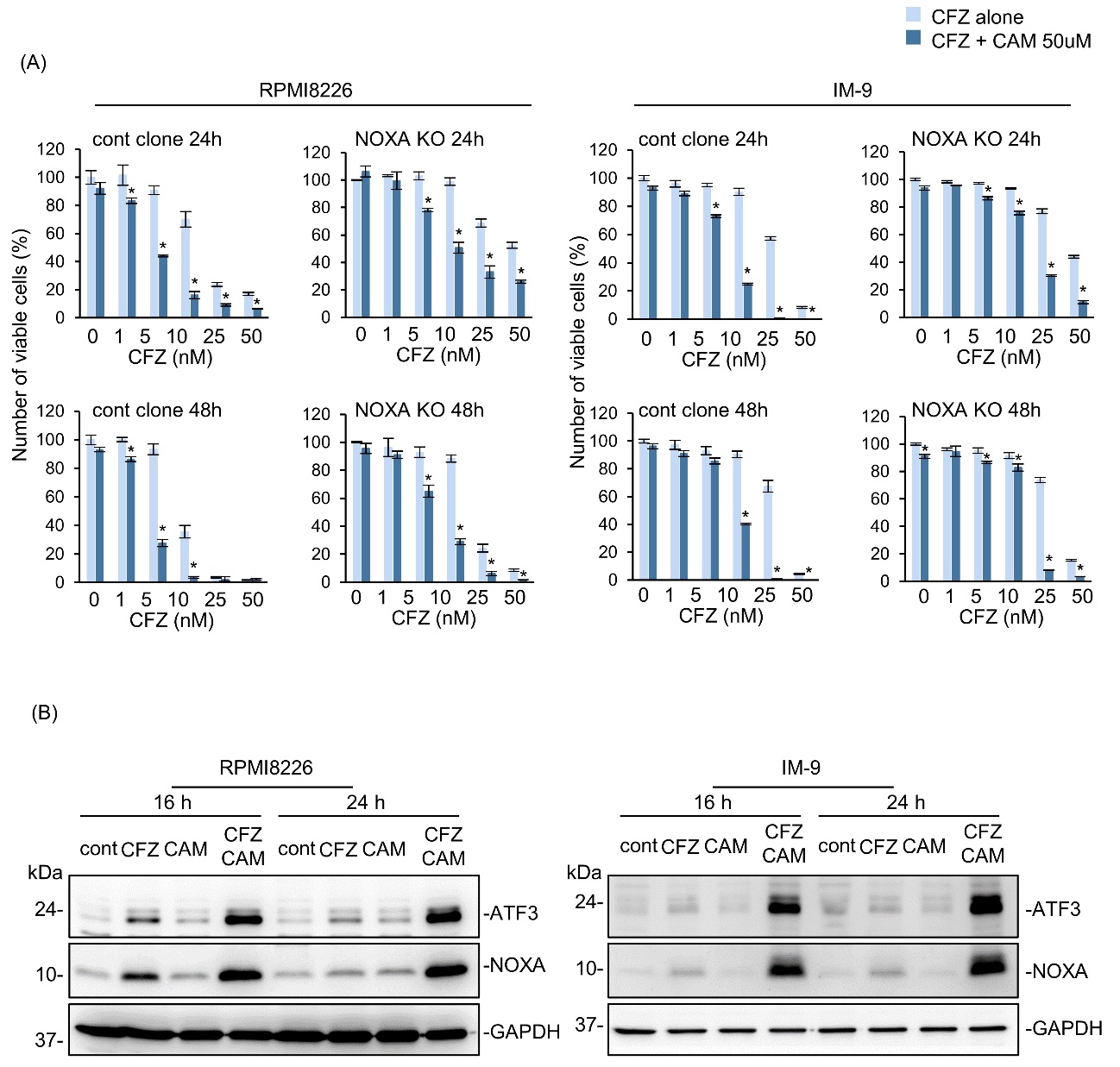


**Supplemental Fig. 2.**

**Combined treatment with carfilzomib (CFZ) and clarithromycin (CAM) enhances ATF3 and NOXA.**

RPMI8226 and IM-9 cells were treated with CFZ (10 nM for RPMI8226, 25 nM for IM-9) and/or CAM (50 μM) for 24 h. Cellular proteins were immunoblotted using anti-ATF3 and anti-NOXA mAbs. Immunoblotting with an anti-GAPDH mAb was performed as an internal control.

**
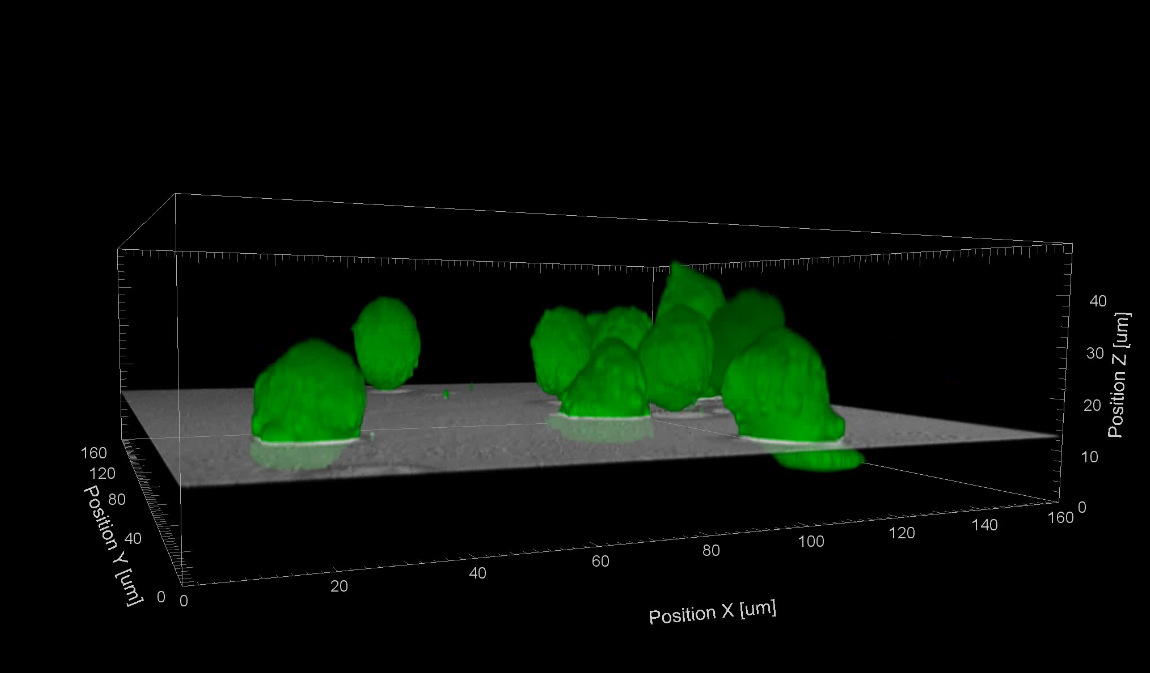
**

**Supplementary Movies (S5 File).**

**Confocal microscopy for monitoring the interaction between MM cells and stromal cells.**

LP101 cells were pre-cultured in CELLview 35-mm glass-bottomed cell culture dishes (#627870; Greiner Bio-One Ltd., UK). The next day, the medium was replaced, and RPMI8226/EGFP cells were seeded. Cells were observed by time-lapse Z-stack imaging using an LSM 700 confocal laser scanning microscope (Carl Zeiss, Germany) with the ZEN 2.3 SP1 Black Edition software (Carl Zeiss). A 3D time-lapse reconstruction was performed using Imaris software (Bitplane AG, Zurich, Switzerland). Notably, MM cells moved onto the surface of the stromal cell layer by untightened cell attachment.
