# Supplementary figures and images for: Clarithromycin overcomes stromal cell-mediated drug resistance against proteasome inhibitors in myeloma cells via autophagy flux blockage leading to high NOXA expression
Source: PLoS One. 2023 Dec 1;18(12):e0295273. doi: 10.1371/journal.pone.0295273 (PMC10691716; doi:10.1371/journal.pone.0295273)

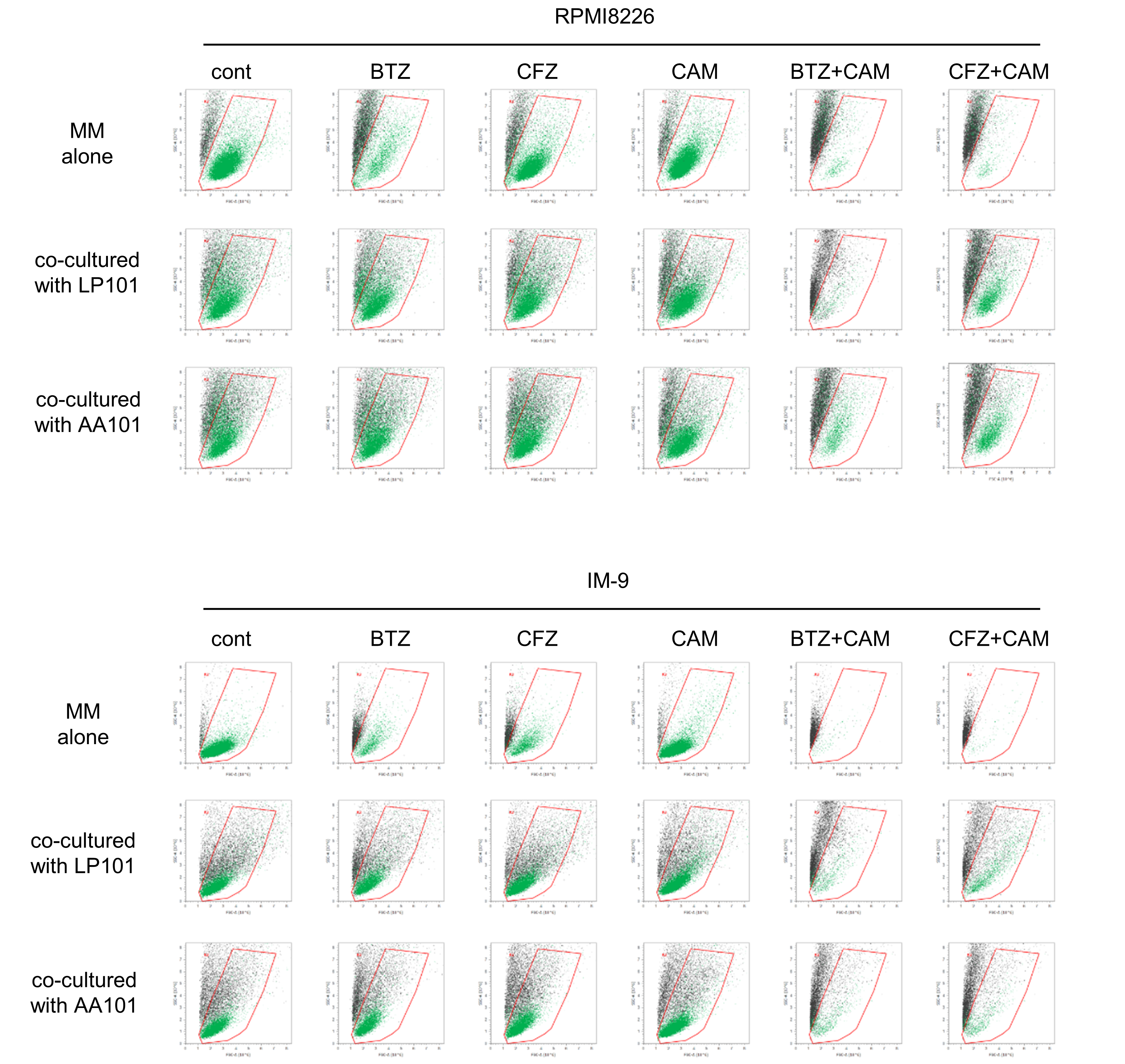

Supplement: S1 Fig — (TIF) [file pone.0295273.s002.tif]

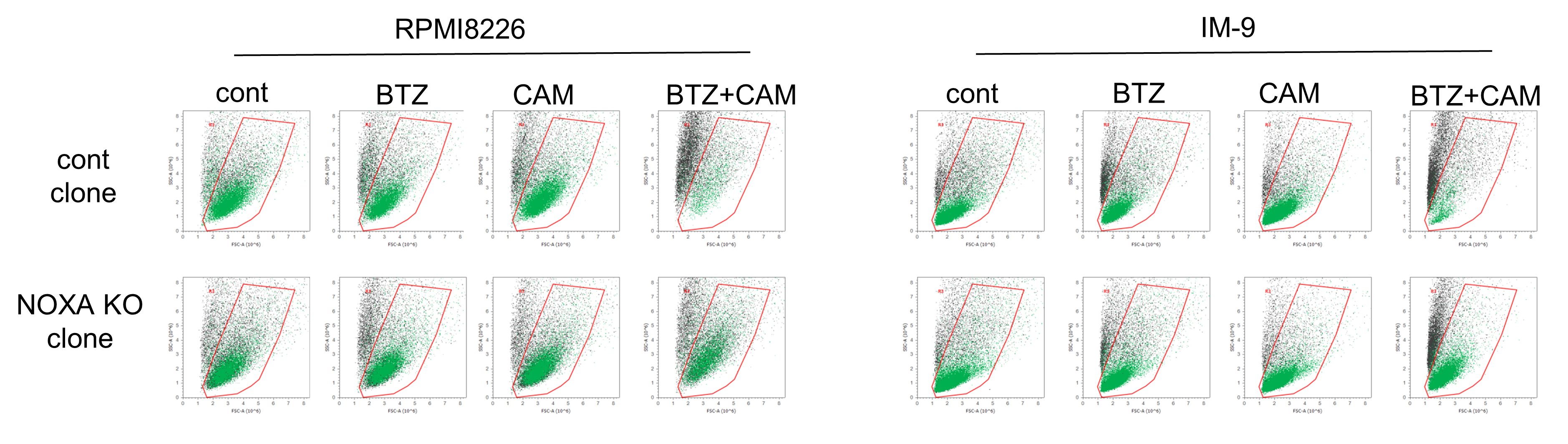

Supplement: S2 Fig — (TIF) [file pone.0295273.s003.tif]
