# Supplementary material for: Clarithromycin overcomes stromal cell-mediated drug resistance against proteasome inhibitors in myeloma cells via autophagy flux blockage leading to high NOXA expression
Source: PLoS One. 2023 Dec 1;18(12):e0295273. doi: 10.1371/journal.pone.0295273 (PMC10691716; doi:10.1371/journal.pone.0295273)
Supplement: S1 Raw images — (PDF) [file pone.0295273.s004.pdf]

Fig.2A

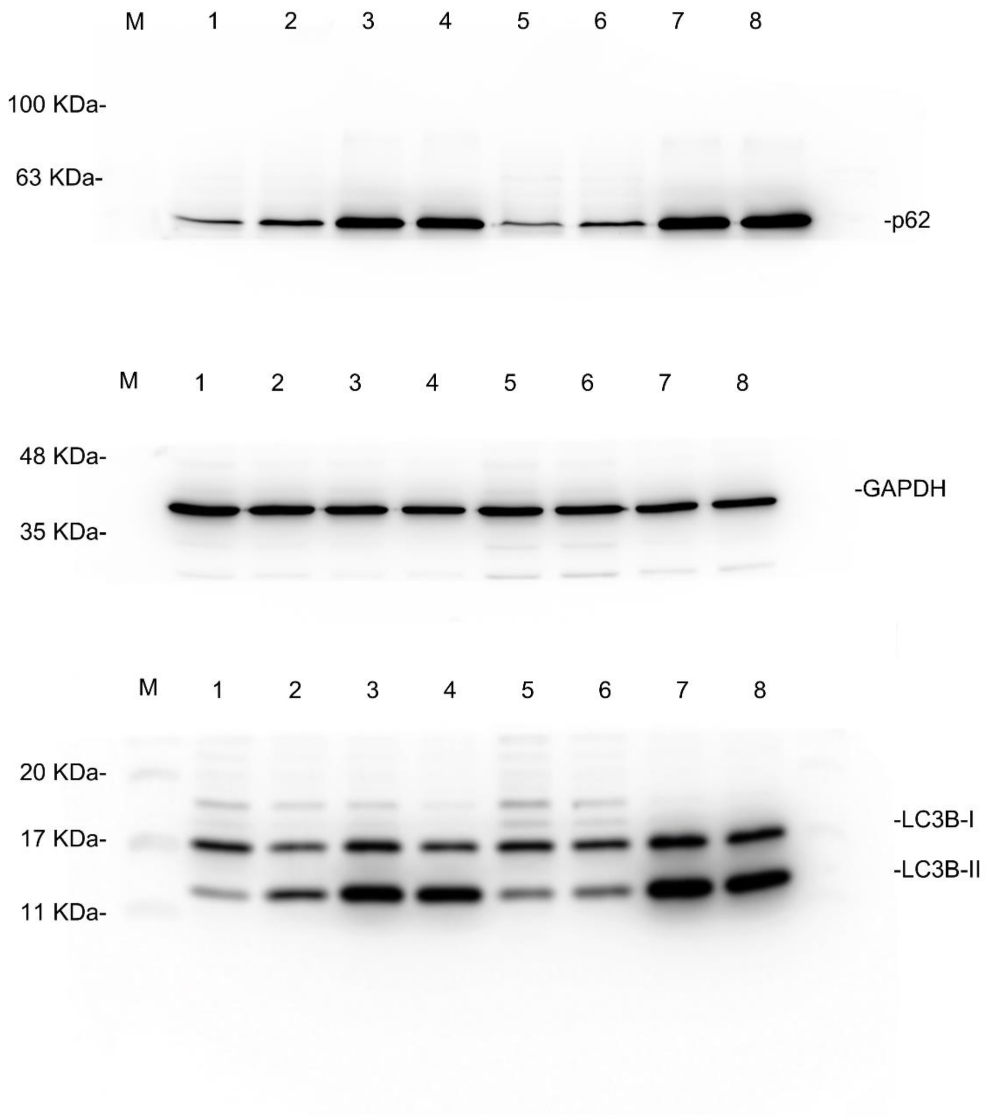

1 RPMI8226 cont.

2 RPMI8226 CAM

3 RPMI8226 BAF

4 RPMI8226 CAM・BAF

5 IM-9 cont.

6 IM-9 CAM

7 IM-9 BAF

8 IM-9 CAM・BAF

Fig.3A ①

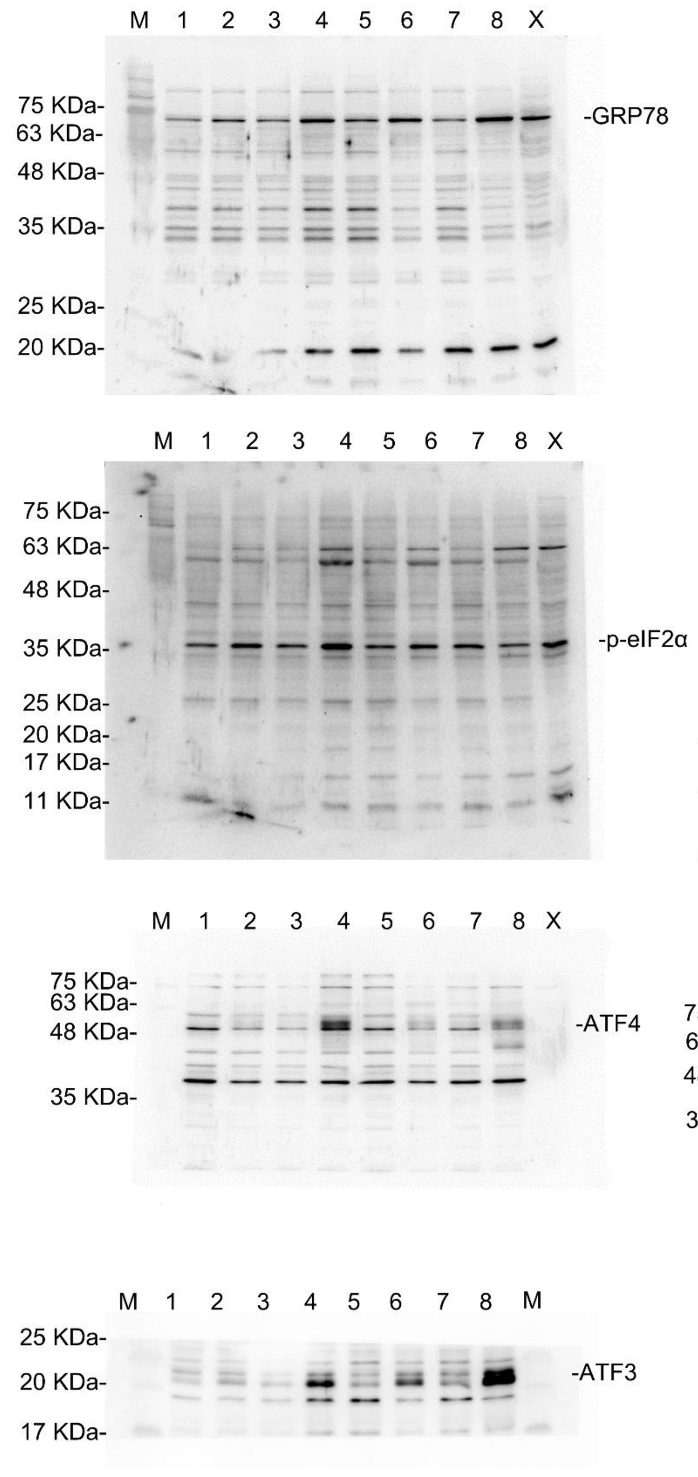

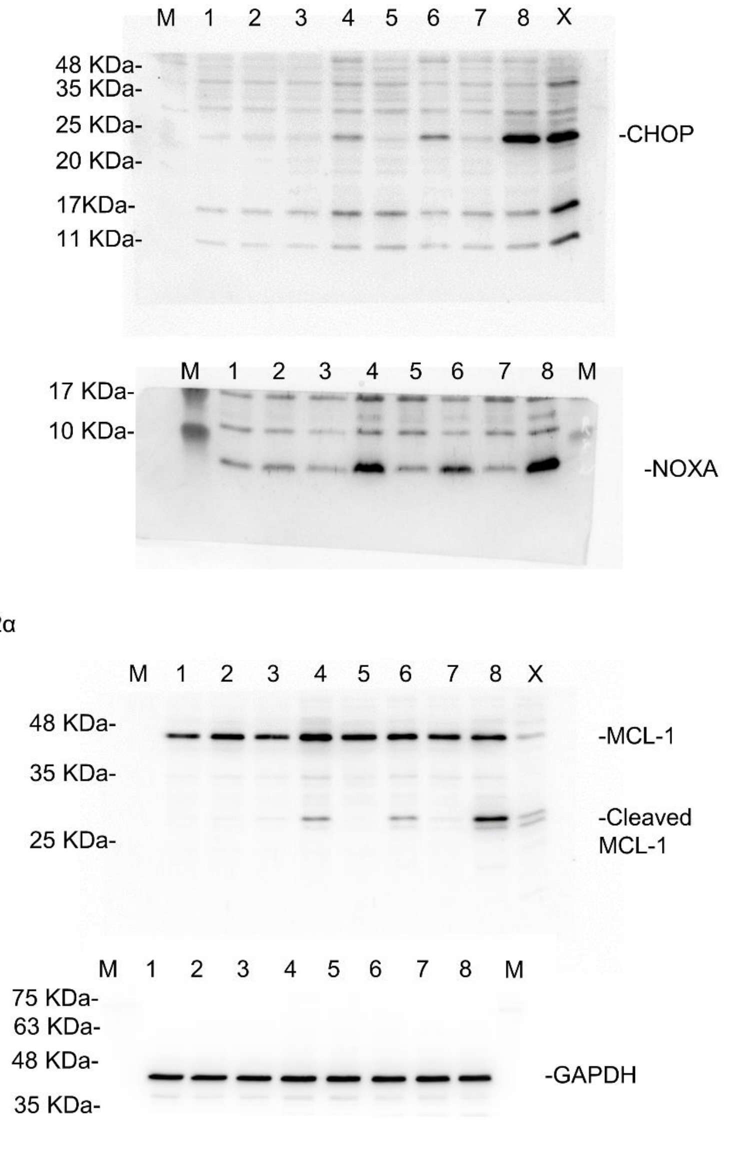

1 RPMI8226, 8h, cont.

2 RPMI8226, 8h, BTZ

3 RPMI8226, 8h, CAM

4 RPMI8226, 8h, BTZ＋CAM

5 RPMI8226, 16h, cont.

6 RPMI8226, 16h, BTZ

7 RPMI8226, 16h, CAM

8 RPMI8226, 16h, BTZ＋CAM

Fig.3A ②

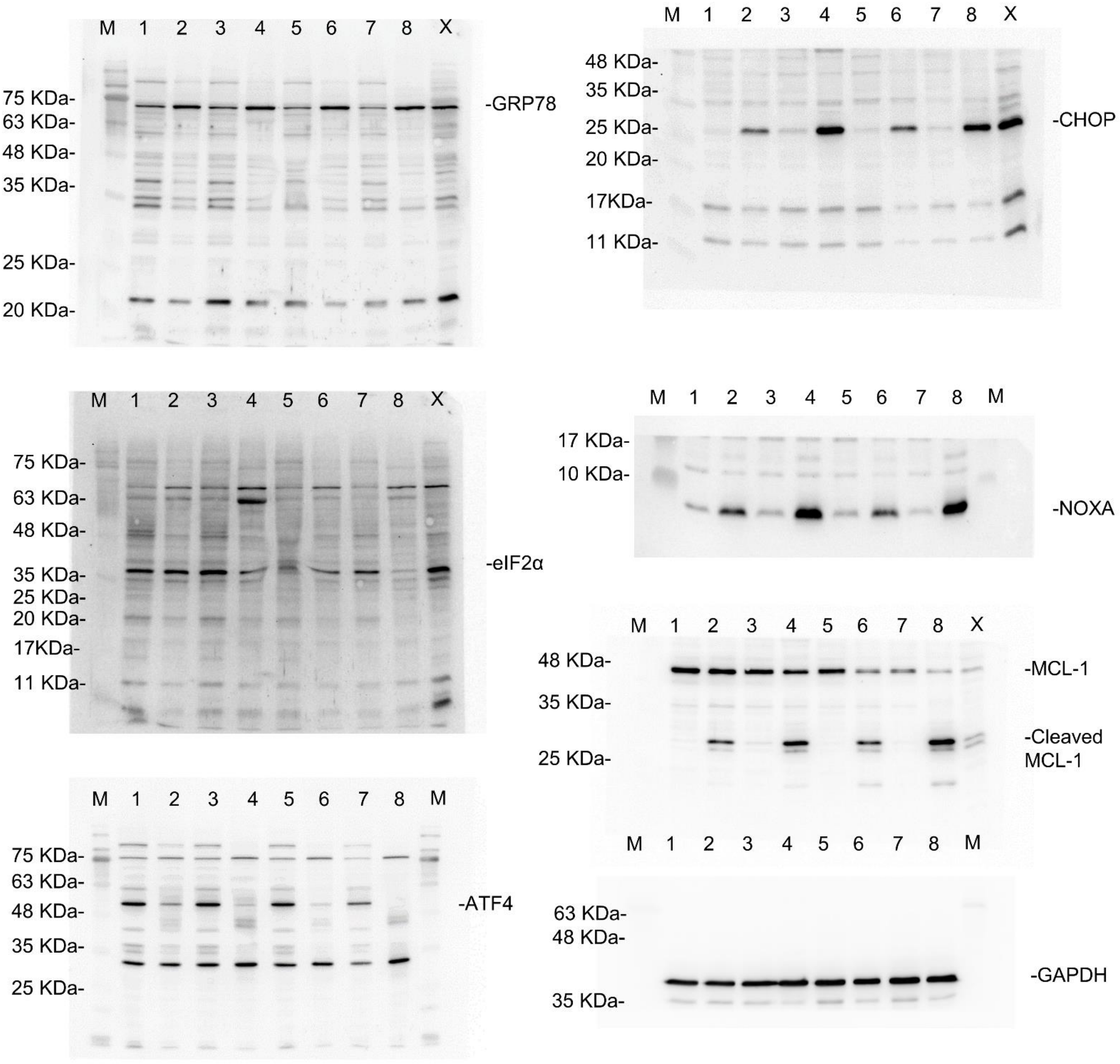

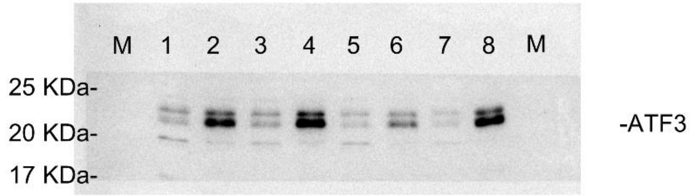

1 RPMI8226, 24h, cont.

2 RPMI8226, 24h, BTZ

3 RPMI8226, 24h, CAM

4 RPMI8226, 24h, BTZ＋CAM

5 RPMI8226, 48h, cont.

6 RPMI8226, 48h, BTZ

7 RPMI8226, 48h, CAM

8 RPMI8226, 48h, BTZ＋CAM

Fig.3A ③

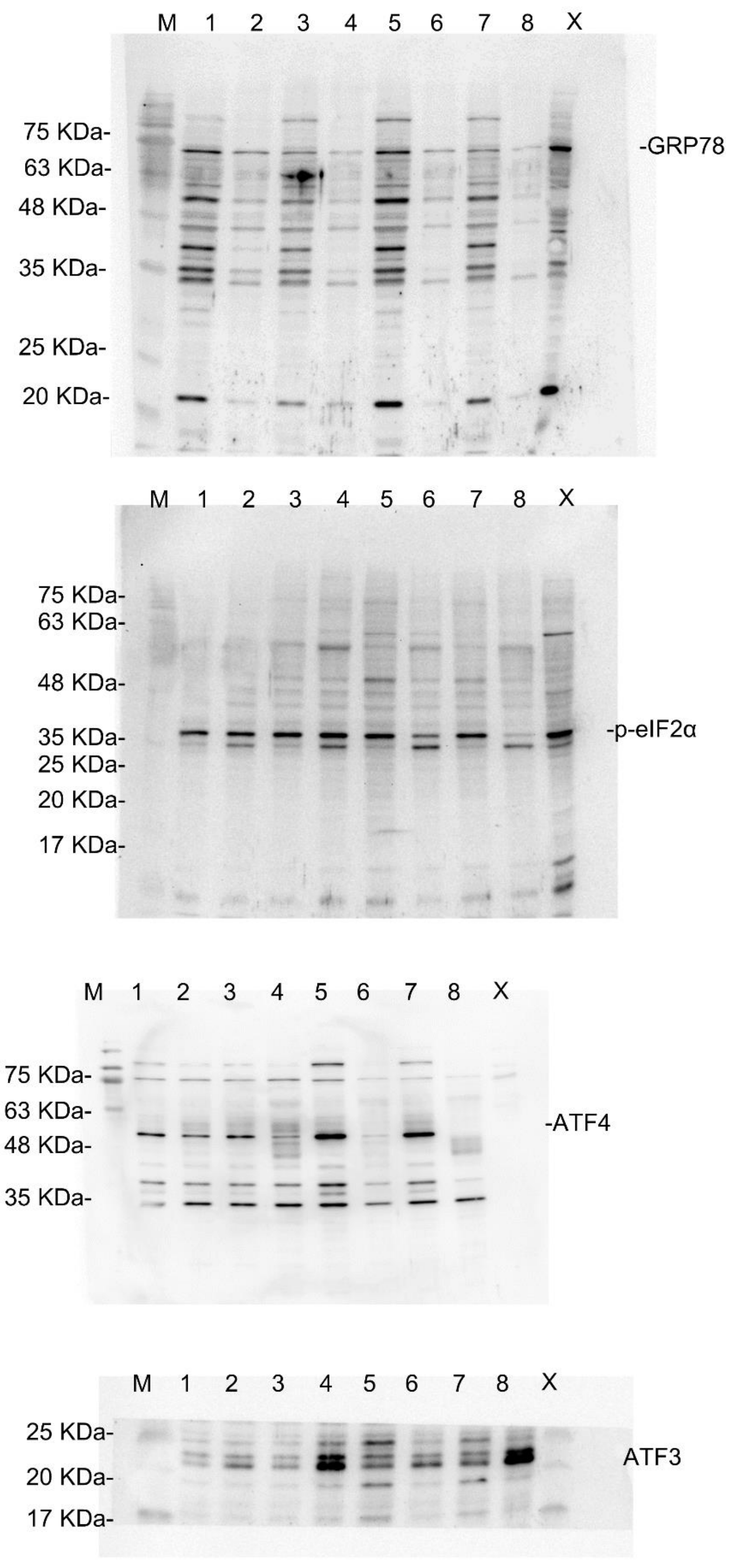

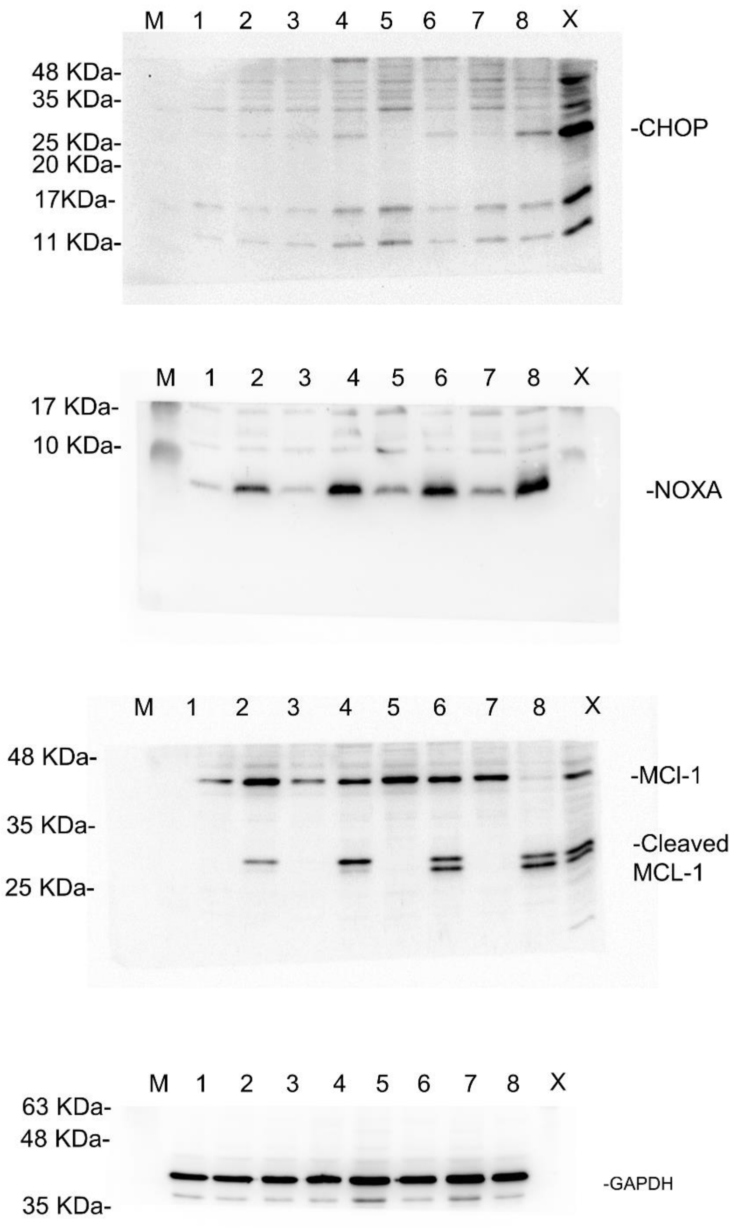

1 IM-9 24h, cont.

2 IM-9, 24h, BTZ

3 IM-9, 24h, CAM

4 IM-9, 24h, BTZ + CAM

5 IM-9, 48h, cont.

6 IM-9, 48h, BTZ

7 IM-9, 48h, CAM

8 IM-9, 48h, BTZ + CAM

Fig.3A ④

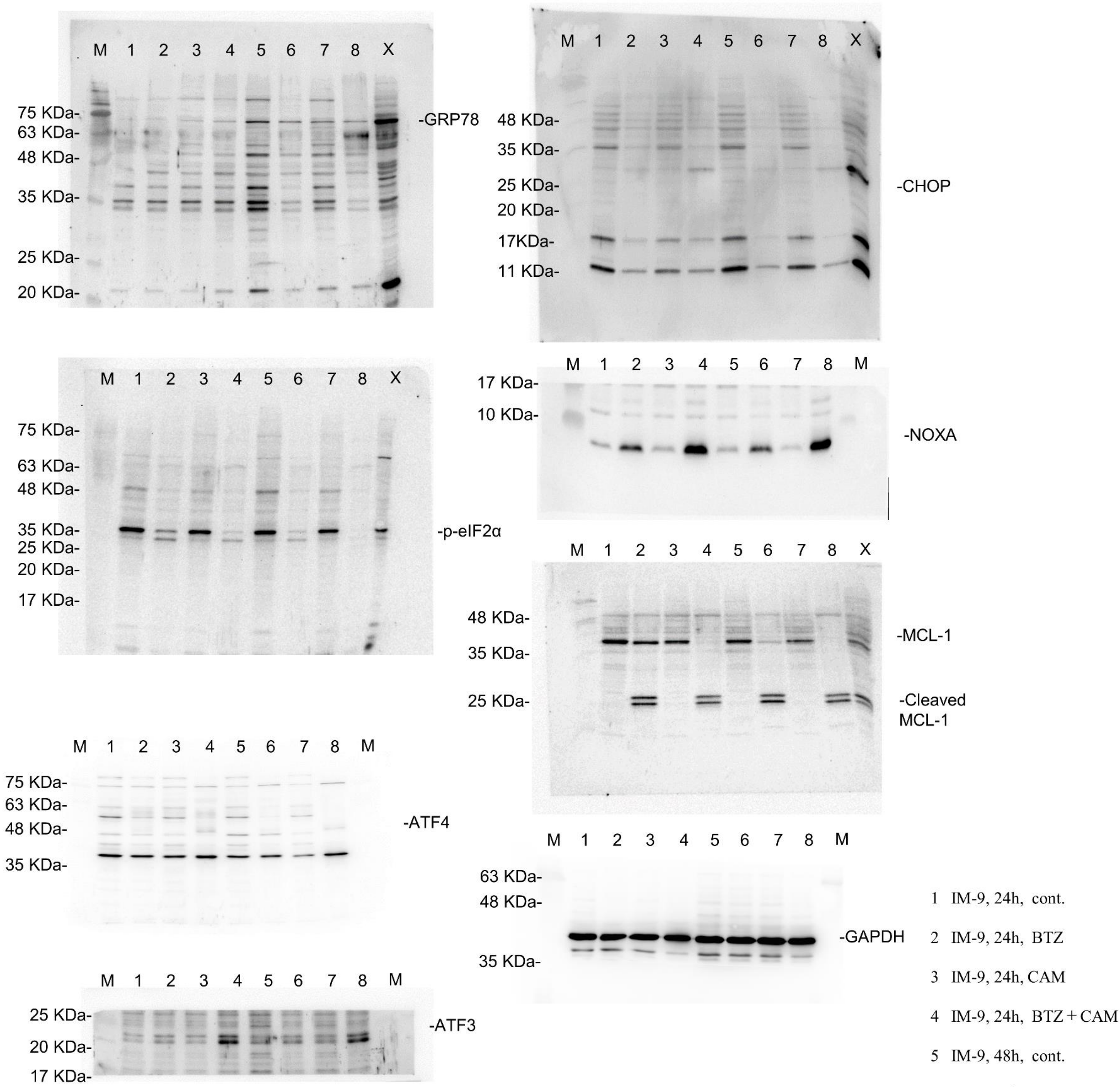

1 IM-9, 24h, cont.

2 IM-9, 24h, BTZ

3 IM-9, 24h, CAM

4 IM-9, 24h, BTZ＋CAM

5 IM-9, 48h, cont.

6 IM-9, 48h, BTZ

7 IM-9, 48h, CAM

8 IM-9, 48h, BTZ＋CAM

Fig.4B ①

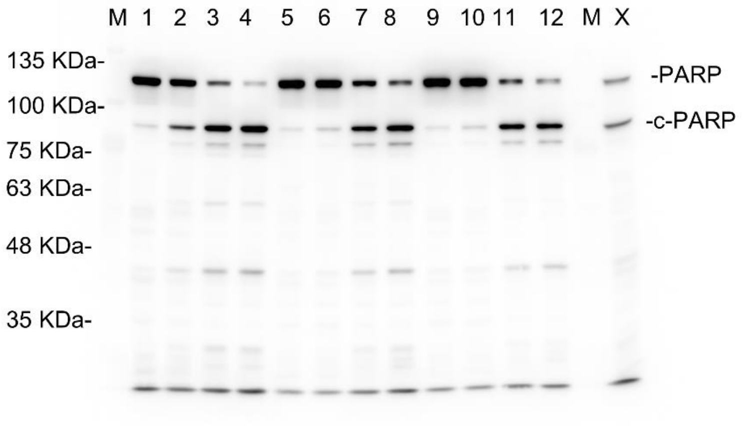

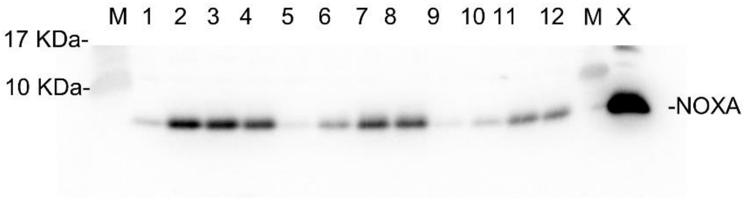

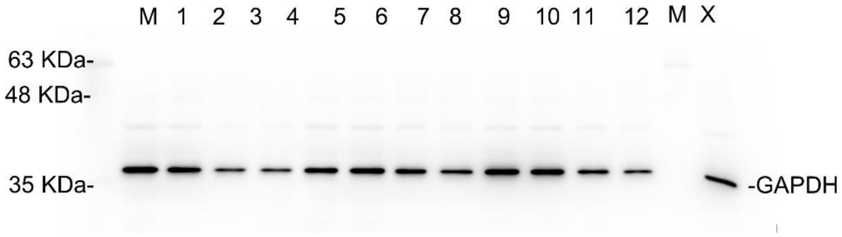

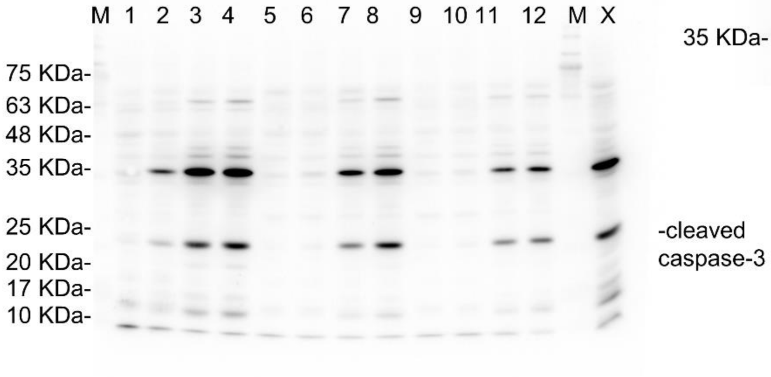

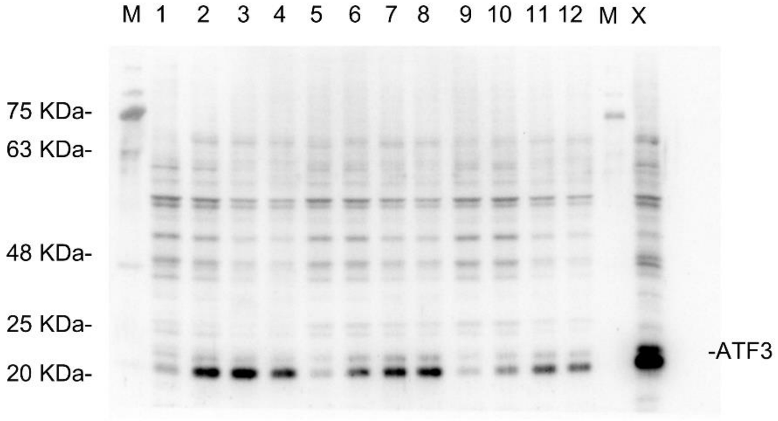

1	RPMI8226 alone	cont
2		BZ 5nM
3		BZ 10nM
4		BZ 15nM
5	RPMI8226 Adhision (-)	cont
6		BZ 5nM
7		BZ 10nM
8		BZ 15nM
9	RPMI8226 Adhision (+)	cont
10		BZ 5nM
11		BZ 10nM
12		BZ 15nM

Fig.4B ②

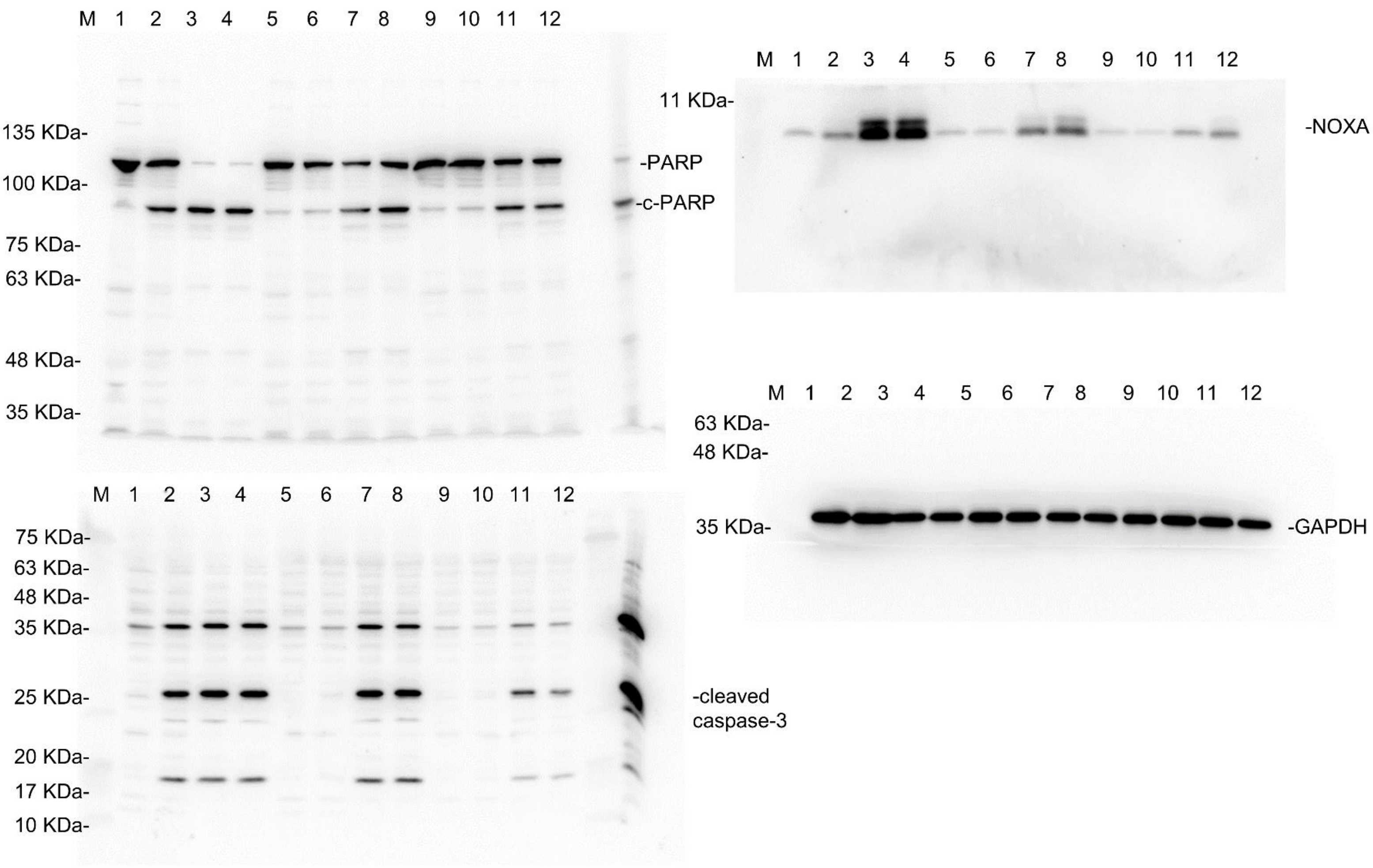

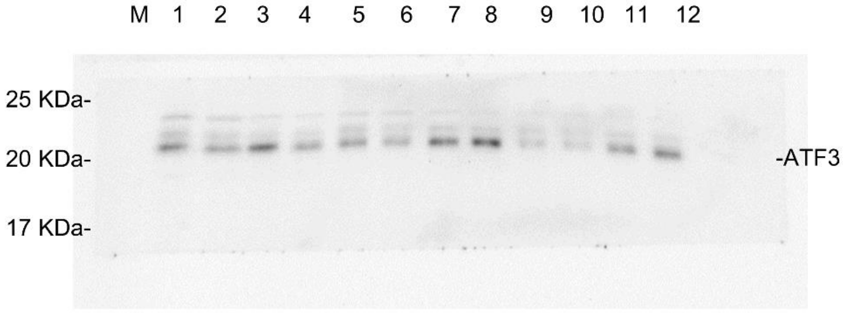

1	IM-9 alone	cont
2		BZ 5nM
3		BZ 10nM
4		BZ 15nM
5	IM-9 Adhision (-)	cont
6		BZ 5nM
7		BZ 10nM
8		BZ 15nM
9	IM-9 Adhision (+)	cont
10		BZ 5nM
11		BZ 10nM
12		BZ 15nM

Fig.4B ③

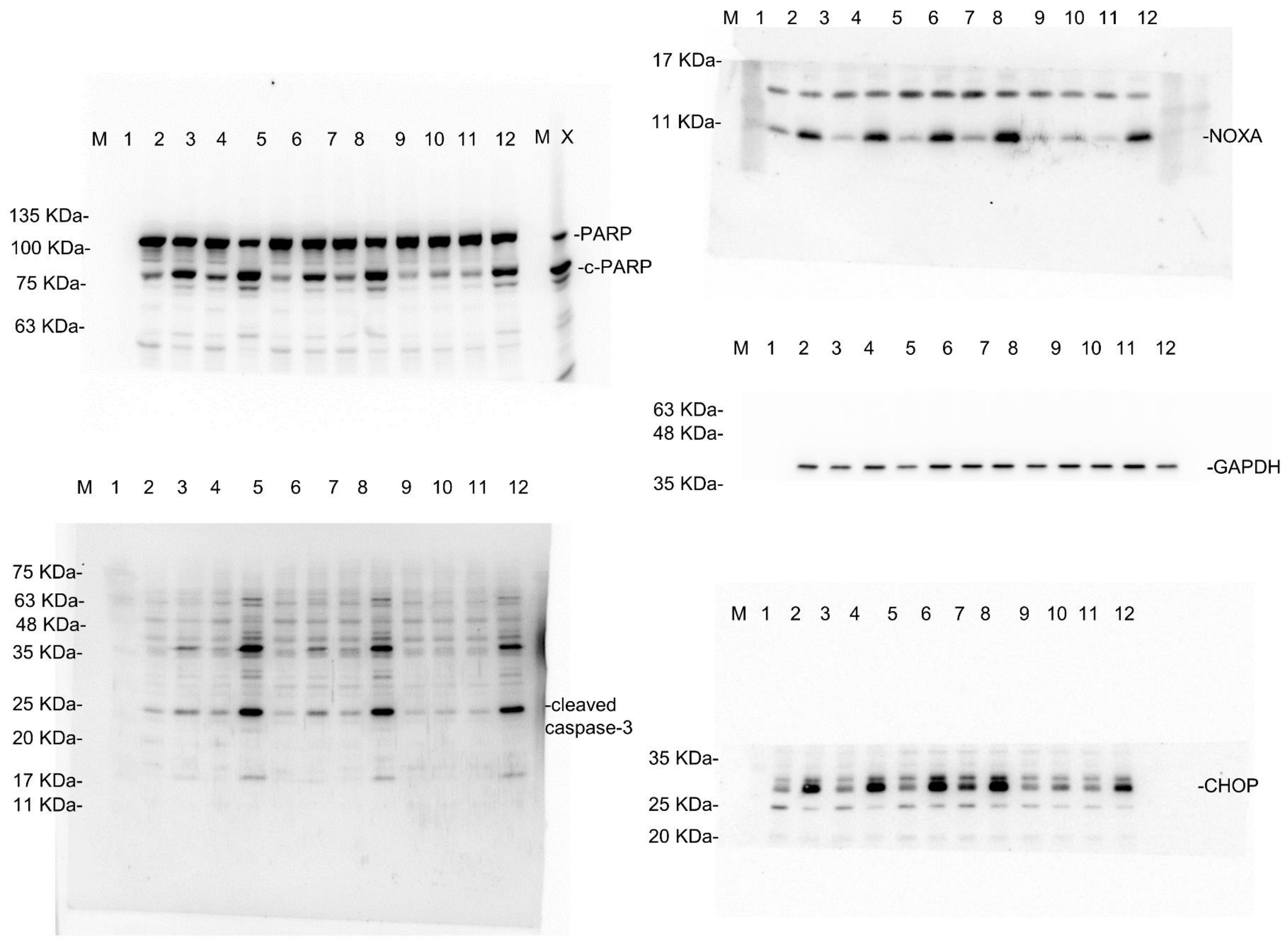

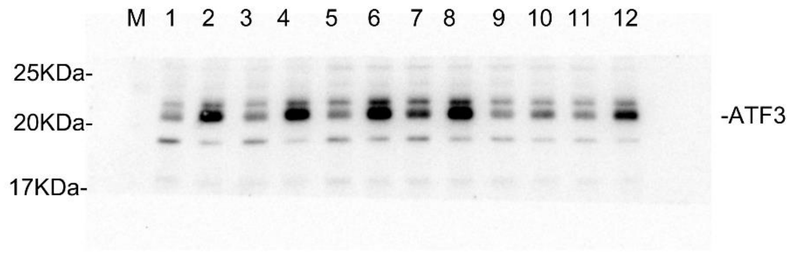

1		cont
2	RPMI8226 cont	BZ
3		CAM
4		BZ+CAM
5		cont
6	RPMI8226 Adhision (-)	BZ
7		CAM
8		BZ+CAM
9		cont
10	RPMI8226 Adhision (+)	BZ
11		CAM
12		BZ+CAM

Fig.4B ④

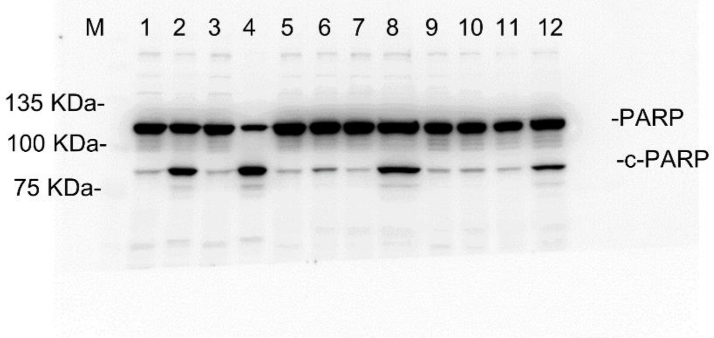

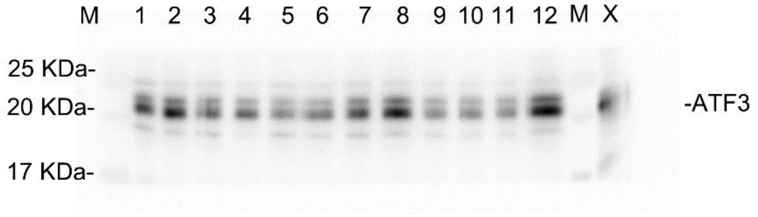

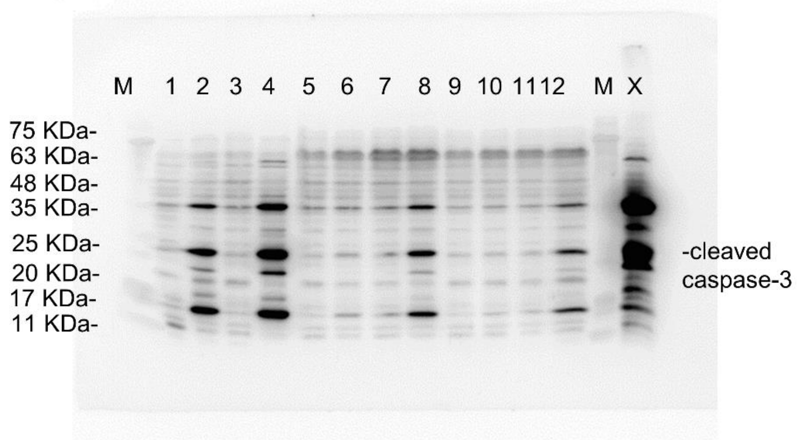

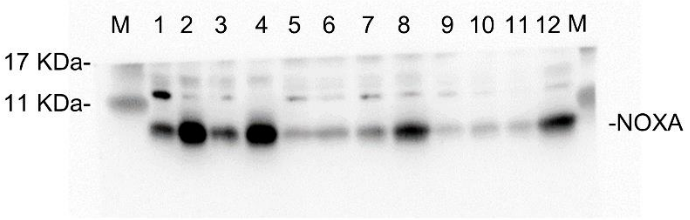

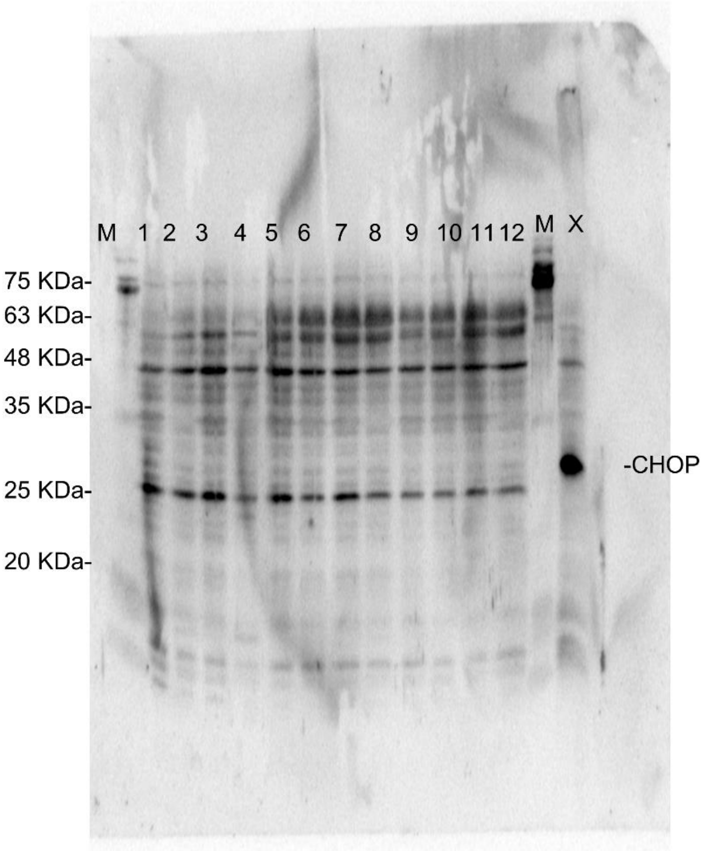

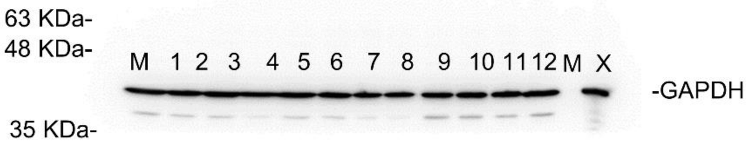

1		cont
2	IM-9 cont	BZ
3		CAM
4		BZ+CAM
5		cont
6	IM-9 Adhision (-)	BZ
7		CAM
8		BZ+CAM
9		cont
10	IM-9 Adhision (+)	BZ
11		CAM
12		BZ+CAM

Fig. 5A

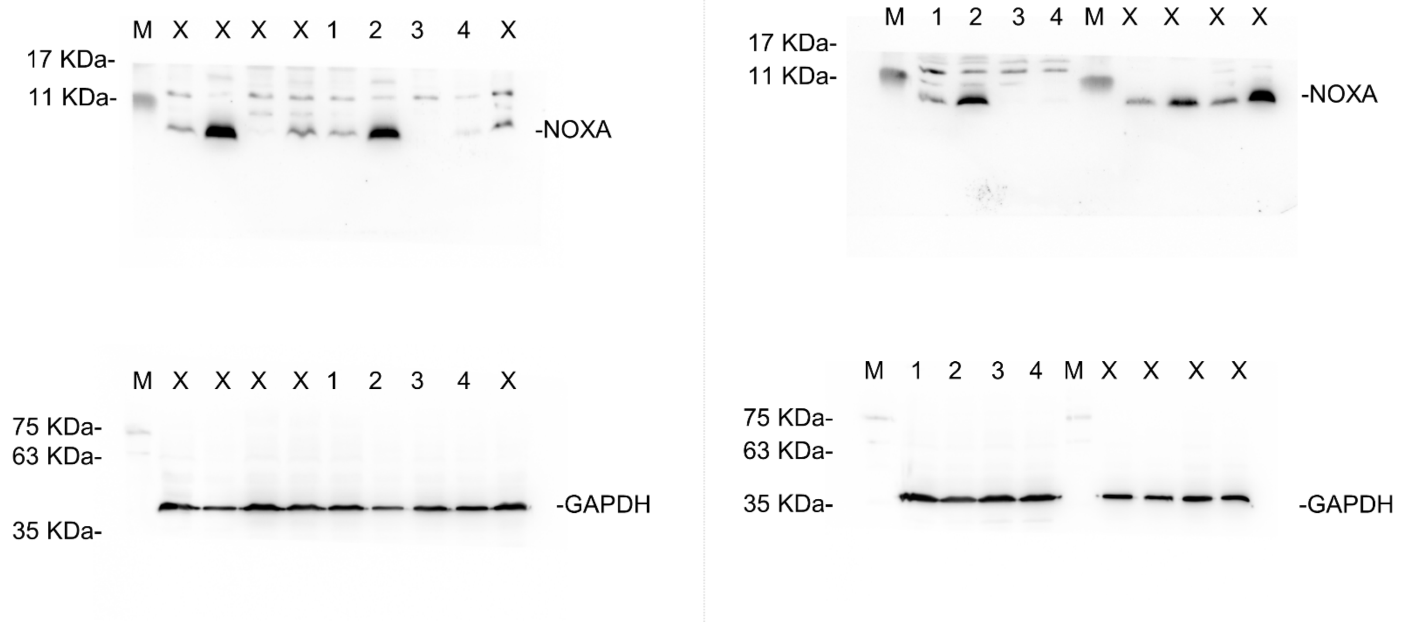

1 RPMI8226/cont, c

2 RPMI8226/cont, BTZ

3 RPMI8226/NOXA KO, c

4 RPMI8226/NOXA KO, BTZ

1 IM-9/cont.

2 IM-9/cont

3 IM-9/NOXA KO

4 IM-9/NOXA KO

Fig. 6A ①

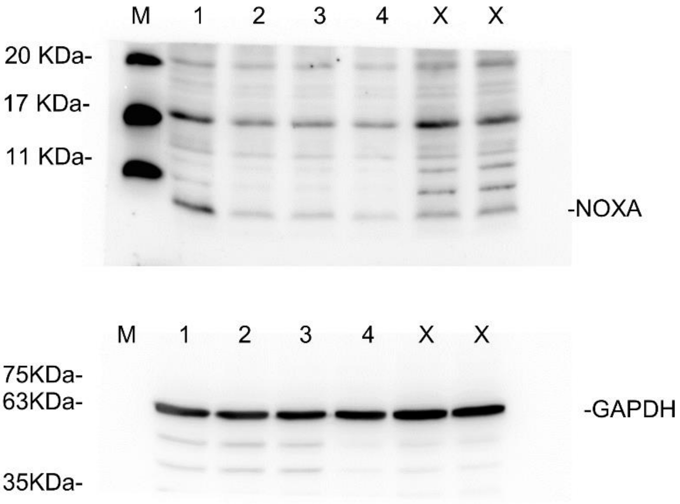

1 CHX 0 h
2 RPMI8226 cont CHX 3 h
3 CHX 5 h
4 CHX 8 h

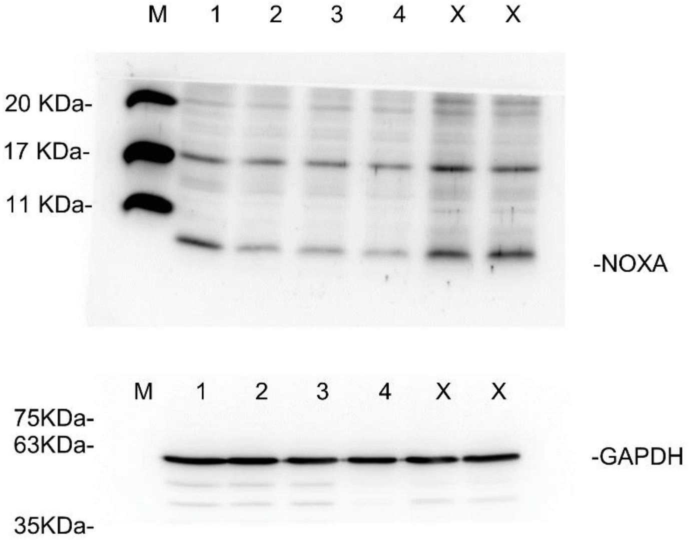

1 CHX 0 h
2 RPMI8226 CAM CHX 3 h
3 CHX 5 h
4 CHX 8 h

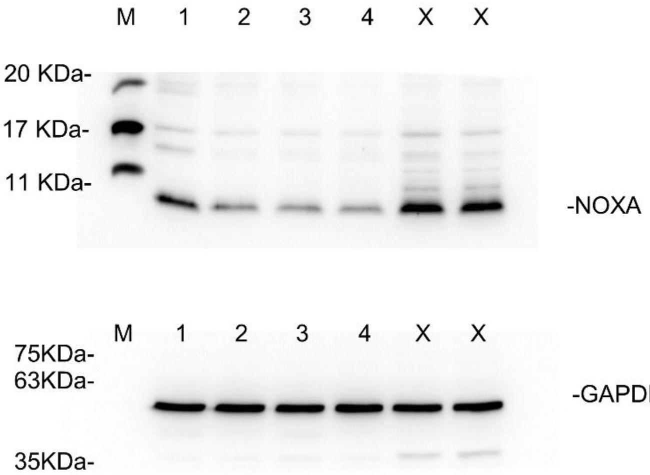

1 CHX 0 h
2 RPMI8226 BZ CHX 3h
3 CHX 5 h
4 CHX 8 h

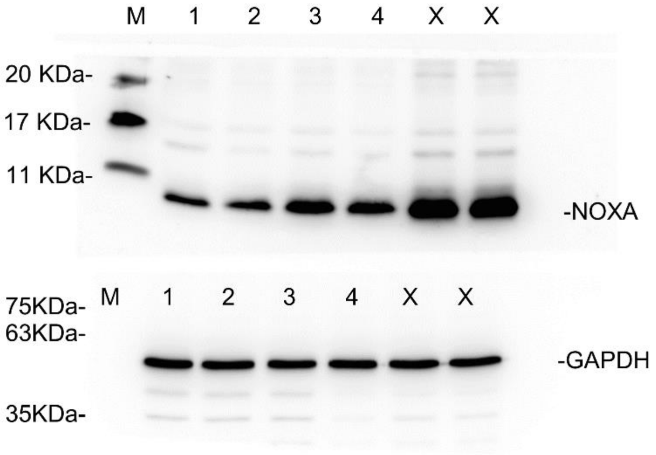

1 CHX 0h
2 RPMI8226 BZ+CAM CHX h
3 CHX h
4 CHX h

Fig. 6A ②

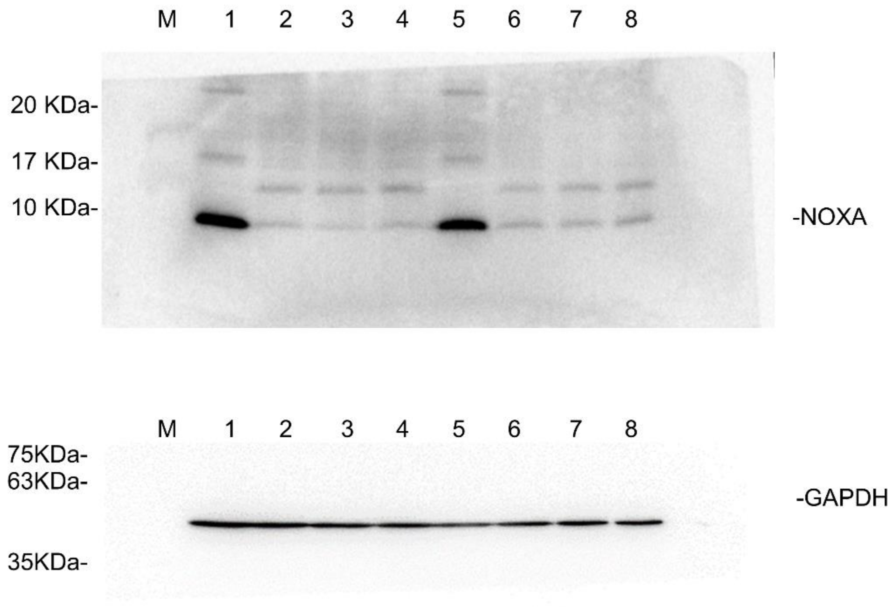

1 IM-9, c, CHX 0 h

2 IM-9, c, CHX 3 h

3 IM-9, c, CHX 5 h

4 IM-9, c, CHX 8 h

5 IM-9, CAM, CHX 0 h

6 IM-9, CAM, CHX 3 h

7 IM-9, CAM, CHX 5 h

8 IM-9, CAM, CHX 8 h

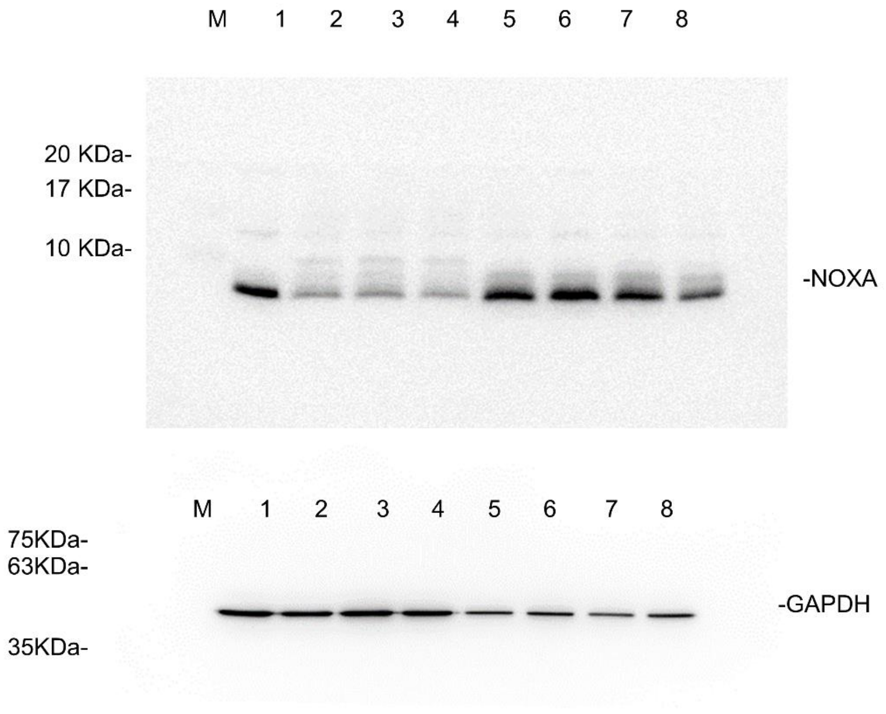

1 IM-9, BZ, CHX 0 h

2 IM-9, BZ, CHX 3 h

3 IM-9, BZ, CHX 5 h

4 IM-9, BZ, CHX 8 h

5 IM-9,BZ+ CAM, CHX 0 h

6 IM-9,BZ+ CAM, CHX 3 h

7 IM-9,BZ+ CAM, CHX 5 h

8 IM-9,BZ+ CAM, CHX 8 h

Fig. 6B

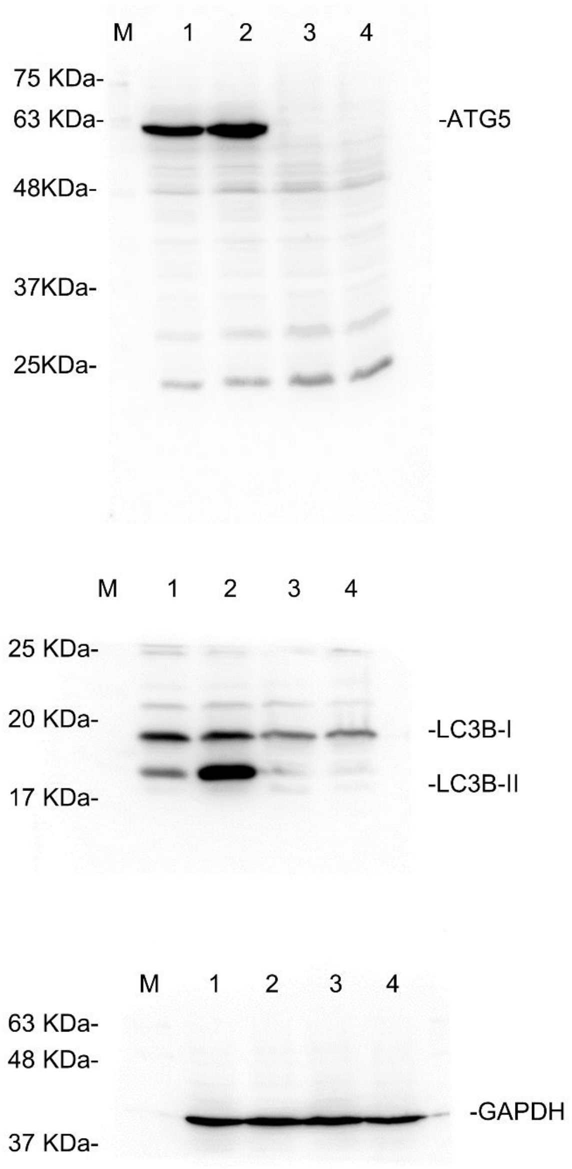

1 293T/cont, c

2 293T/cont, BAF

3 293T/ATG5 KO, c

4 293T/ATG5 KO, BAF

Fig. 6C

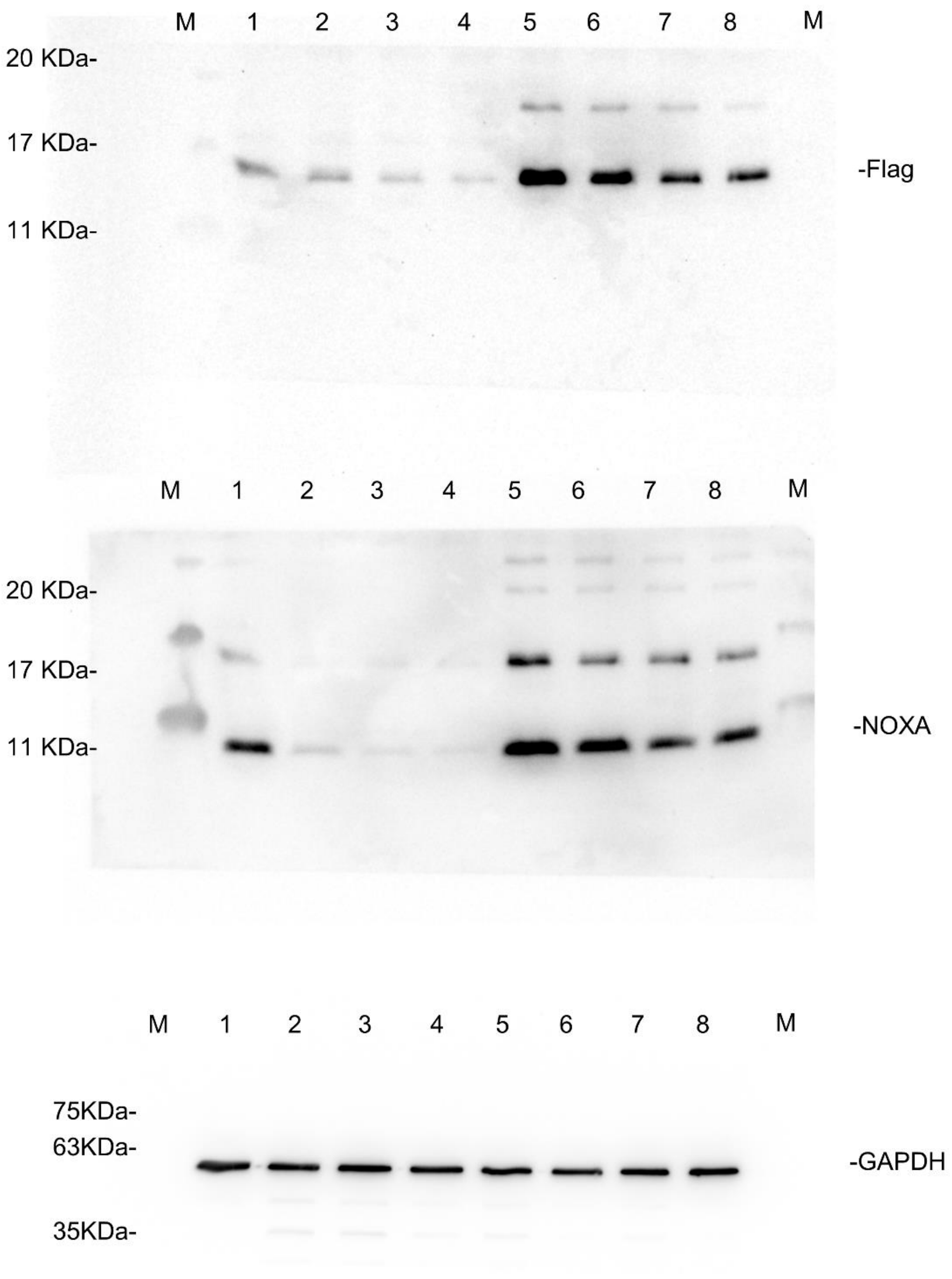

1 293T/cont, CHX 0h

2 293T/cont, CHX 3h

3 293T/cont, CHX 5h

4 293T/cont, CHX 8h

5 293T/ATG5 KO, CHX 0h

6 293T/ATG5 KO, CHX 3h

7 293T/ATG5 KO, CHX 5h

8 293T/ATG5 KO, CHX 8h

Fig. 6D ①

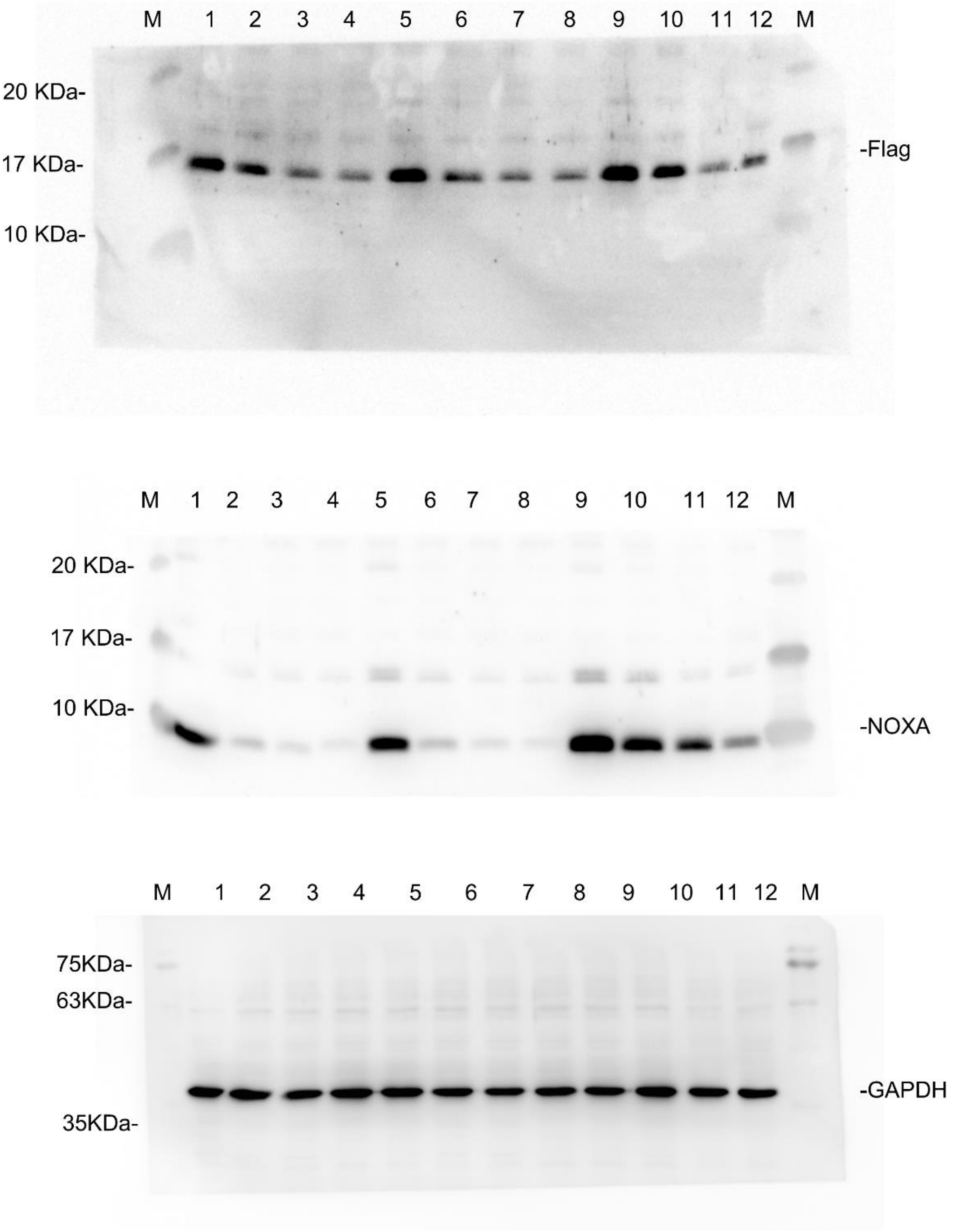

1 293T/cont, c, CHX 0h

2 293T/cont, c, CHX 3h

3 293T/cont, c, CHX 5h

4 293T/cont, c, CHX 8h

5 293T/cont, BZ 5nM, CHX 0h

6 293T/cont, BZ 5nM, CHX 3h

7 293T/cont, BZ 5nM, CHX 5h

8 293T/cont, BZ 5nM, CHX 8h

9 293T/cont, BZ 10nM, CHX 0h

10 293T/cont, BZ 10nM, CHX 3h

11 293T/cont, BZ 10nM, CHX 5h

12 293T/cont, BZ 10nM, CHX 8h

Fig. 6D ②

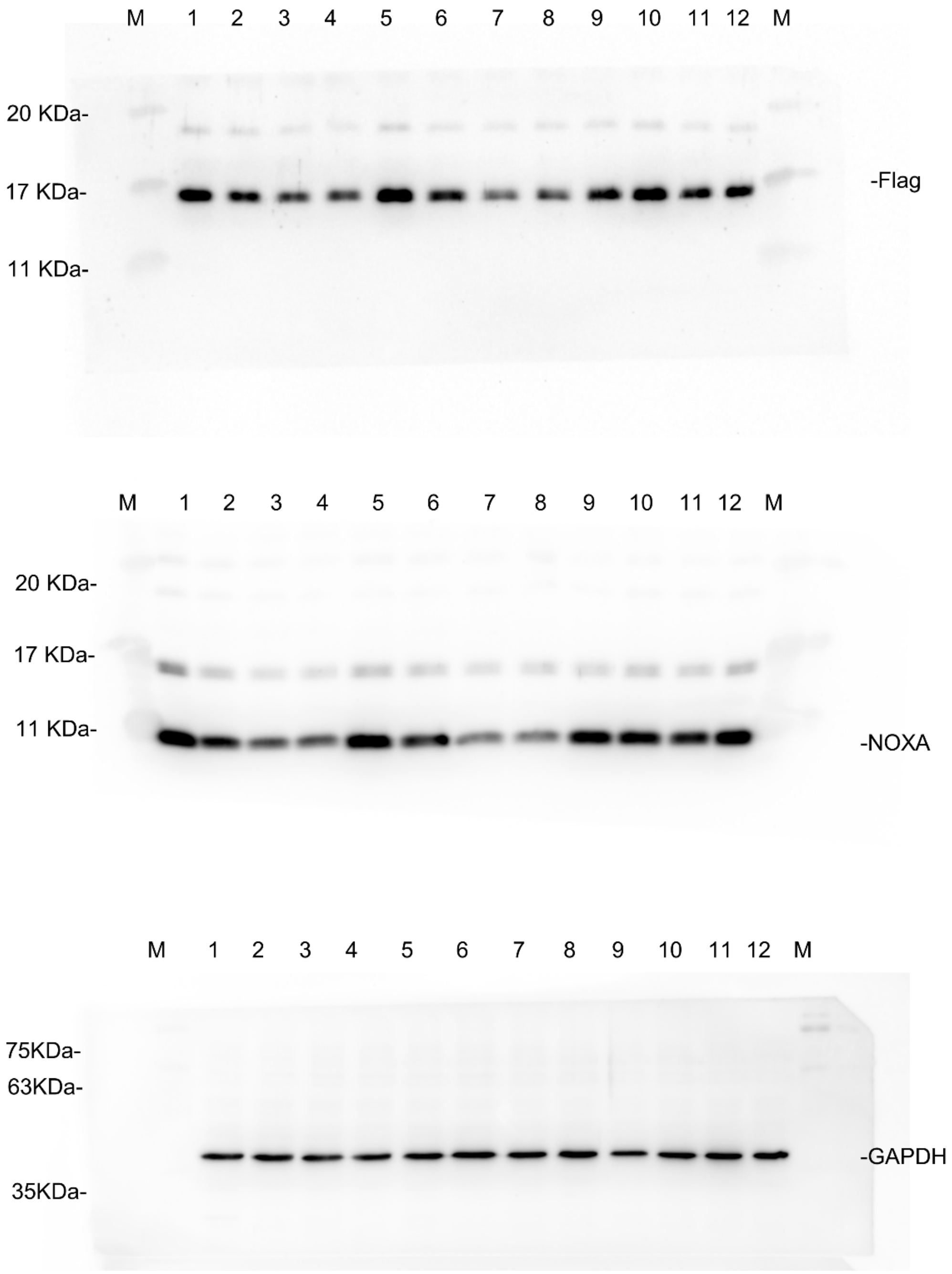

1 293T/ATG5 KO, c, CHX 0h

2 293T/ATG5 KO, c, CHX 3h

3 293T/ATG5 KO, c, CHX 5h

4 293T/ATG5 KO, c, CHX 8h

5 293T/ATG5 KO, BZ 5nM, CHX 0h

6 293T/ATG5 KO, BZ 5nM, CHX 3h

7 293T/ATG5 KO, BZ 5nM, CHX 5h

8 293T/ATG5 KO, BZ 5nM, CHX 8h

9 293T/ATG5 KO, BZ 10nM, CHX 0h

10 293T/ATG5 KO, BZ 10nM, CHX 3h

11 293T/ATG5 KO, BZ 10nM, CHX 5h

12 293T/ATG5 KO, BZ 10nM, CHX 8h

S Fig.2

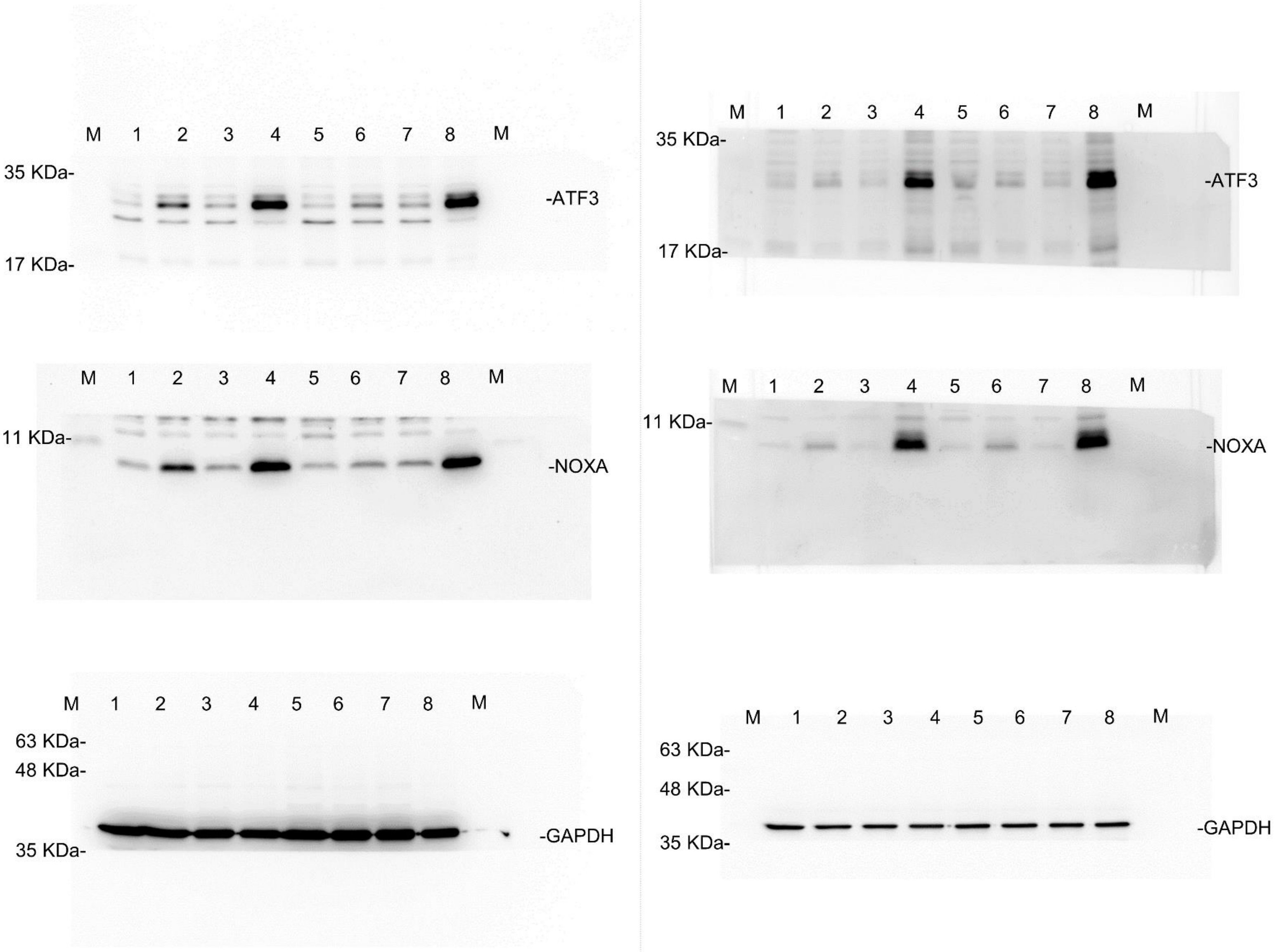

1 RPMI8226, 16h, cont.

2 RPMI8226, 16h, CFZ

3 RPMI8226, 16h, CAM

4 RPMI8226, 16h, CFZ＋CAM

5 RPMI8226, 24h, cont.

6 RPMI8226, 24h, CFZ

7 RPMI8226, 24h, CAM

8 RPMI8226, 24h, CFZ＋CAM

1 IM-9, 16h, cont.

2 IM-9, 16h, CFZ

3 IM-9, 16h, CAM

4 IM-9, 16h, CFZ＋CAM

5 IM-9, 24h, cont.

6 IM-9, 24h, CFZ

7 IM-9, 24h, CAM

8 IM-9, 24h, CFZ＋CAM
