# Supplementary material for: Clarithromycin overcomes stromal cell-mediated drug resistance against proteasome inhibitors in myeloma cells via autophagy flux blockage leading to high NOXA expression
Source: PLoS One. 2023 Dec 1;18(12):e0295273. doi: 10.1371/journal.pone.0295273 (PMC10691716; doi:10.1371/journal.pone.0295273)
Supplement: S1 Table — (DOCX) [file pone.0295273.s006.docx]

| **Table S1. Primer sequences for real-time PCR** | |  |  |
| --- | --- | --- | --- |
|  |  |  |  |
| **Gene** | **Forward (5'-3')** | **Reverse (5'-3')** |  |
| human GRP78 | CCTAGCTGTGTCAGAATCTCCATCC | GTTTCAATGTCACCATCCAAGATCC |  |
| human CHOP | AAATCAGAGCTGGAACCTGAGGA | CCATCTCTGCAGTTGGATCAGTC |  |
| human ATF3 | CCTCTGCGCTGGAATCAGTC | TTCTTTCTCGTCGCCTCTTTTT |  |
| human ATF4 | CTGCCCGTCCCAAACCTTAC | GCCCTCTTCTTCTGGCGGTA |  |
| human NOXA | GGAGATGCCTGGGAAGAAG | CCTGAGTTGAGTAGCACACTCG |  |
| human Mcl-1 | AAGCCAATGGGCAGGTCT | TGTCCAGTTTCCGAAGCAT |  |
| human GAPDH | GCACCGTCAAGGCTGAGAAC | TGGTGAAGACGCCAGTGGA |  |
