# Supplementary material for: Clarithromycin overcomes stromal cell-mediated drug resistance against proteasome inhibitors in myeloma cells via autophagy flux blockage leading to high NOXA expression
Source: PLoS One. 2023 Dec 1;18(12):e0295273. doi: 10.1371/journal.pone.0295273 (PMC10691716; doi:10.1371/journal.pone.0295273)
Supplement: Attachment — Submitted filename: Response to Reviewers20230922.docx [file pone.0295273.s007.docx]

**Response to Reviewers：**

**To Reviewer #1:**

Thank you for your valuable comments. Your comments were extremely helpful for us. We revised our manuscript as much as possible.

**Reviewer's comments.:**

**My overall impression is that this manuscript provides very important information for researchers and medical doctors treating MM. It should be appreciated in respect of developing the study of MM and the treatments for RRMM.**

**Requested Revisions:**

**The text seems to be somewhat complicated and difficult to understand. The manuscript could be more precise and compacted. The use of supplemental figures could be avoided. Plain figures, if possible, could appear successively in the text without supplemental ones. Numbering of references could be in conformity to the submission instruction.**

**Response:**

Thank you for your careful reading and helpful comment. We are very pleased the reviewer commented the significance of this study. Following your suggestion, we revised our manuscript as much as possible the Results section to be more compact and reduced the number of words.

We moved the SFig. 3, showing that CAM inhibits autophagy flux to plane Fig.2A,

and SFig. 2, showing the combined effect of CFZ and CAM, moved to plane Fig. 5.

Thank you for giving us the opportunity to strengthen our manuscript with your valuable comments and queries. These revisions persuade you to accept our submission.

**To Reviewer #2:**

Thank you for your valuable comments. Your comments were extremely helpful for us. We revised our manuscript as much as possible.

**1) The IM-9 cell line used in this study is a lymphoblastoid cell line, not a MM cell line ([Pellat-Deceunynk el al., Blood. 86(10): 4001, 1995](https://ashpublications.org/blood/article/131/1/154/107690/Pellat-Deceunynk-C-Amiot-M-Bataille-R-et-al-Human))**

**Response:**

Thank you for your careful reading and helpful comment. IM-9 cells were derived from a multiple myeloma patient, but IM-9 cells appear to be B-lymphoblastoid cells rather than myeloma-derived cells ([PMID: 7579375](https://www.sciencedirect.com/science/article/pii/S000649712063261X?via%3Dihub)).

However, IM-9 cells secrete IgG and retains plasmacytoma characteristics.

(<https://www.atcc.org/products/ccl-159>)

IM-9 cells are　use in MM research as a MM model. ([PMID: 11494147](https://www.nature.com/articles/1204623)), ([PMID: 11468181](https://ashpublications.org/blood/article/98/3/795/53252/TRAIL-Apo2L-ligand-selectively-induces-apoptosis)), ([PMID: 8562936](https://www.sciencedirect.com/science/article/pii/S0006497120653630?via%3Dihub)), ([PMID: 35503759](https://journals.plos.org/plosone/article?id=10.1371/journal.pone.0265958))

In addition, we have always been examined in parallel with RPMI 8226 cells, and similar results have been obtained with the two cell lines. Thus, we believe the universality of our study has been preserved.

Following your advice, the manuscript has been revised as follows.

Materials and methods: Two cell lines, IM-9 and RPMI8226, derived from MM patients were obtained from ATCC. IM-9 has the lymphoblastic features with secreting IgG.

**2) To my knowledge, Noxa is not directly degraded by autophagy, but only by the proteasome (Craxton et al., Cell Death and Differentiation 19: 1424–1434, 2012). So, the hypothesis provided by the authors is not reliable.**

**Response:**

Thank you for your valuable comments. Your comments were extremely helpful for us.

NOXA is a short-lived protein and it is known to be degraded by the proteasome ([PMID: 28670929](https://pubmed.ncbi.nlm.nih.gov/28670929/)) ([PMID: 24457957](https://www.ncbi.nlm.nih.gov/pmc/articles/PMC4040662/)). Wang et al. reported that NOXA is ubiquitinated in K35/K41/K48 and introduced to p62-mediated autophagosome incorporation for autophagy degradation in human non-small cell lung cancer (NSCLC) and colorectal cancer cell lines ([PMID: 29758299](https://pubmed.ncbi.nlm.nih.gov/29758299/)). Heine et al. reported that the combined treatment of BTZ plus autophagy inhibitor enhances BTZ-induced cell death via blocking autophagic degradation of NOXA in mantle cell lymphoma ([PMID: 30180865](https://jhoonline.biomedcentral.com/articles/10.1186/s13045-018-0657-6)). In a mouse model, NOXA has an FNLV sequence, which is known as the LC3-interacting region (LIR) motif, suggesting that NOXA is engulfed into autophagosomes via LC3 interaction and subsequently degraded by autophagy ([PMID: 27670885](https://www.embopress.org/doi/full/10.15252/embr.201642565)). Recently, we reported that in lung cancer cell lines, NOXA was upregulated via p53 after treatment with DNA-damaging drugs, and NOXA expression was further enhanced in the presence of azithromycin, which has a potent autophagy inhibitory effect ([PMID: 34051014](https://onlinelibrary.wiley.com/doi/10.1111/cas.14992)).

Many studies have reported autophagic degradation of NOXA. Therefore, we investigated the autophagic degradation of NOXA in MM cells. Cycloheximide chase analysis showed that combined treatment with BTZ plus CAM prolonged the half-life of NOXA (Fig. 6A). Furthermore, we attempted to establish a FLAG-tagged NOXA expression vector driven by the CMV promoter to exclude other transcriptional regulators and transfected this plasmid into MM cell lines. ATG5-KO 293T cells, the addition of BTZ alone maintained high FLAG-NOXA expression compared with 293T control cells (Fig. 6D). These data indicate that NOXA is degraded by autophagy and proteasomes.

Autophagic degradation of NOXA reported in mantle cell lymphoma by Heine et al. was not described in the reference. This reference is helpful in the discussion. The manuscript was revised and this reference newly cited. ([PMID: 30180865](https://jhoonline.biomedcentral.com/articles/10.1186/s13045-018-0657-6))

**3) Most of results of the present article were already reported by authors in previous works (IJO 46: 474-486, 2015 and IJO 42: 1541 1550, 2013)**

**Response:**

Thank you for your careful reading and helpful comment. The comments were extremely important and helpful for us. We previously reported that macrolide antibiotics such as CAM inhibit the autophagy flux and combined treatment of BTZ plus CAM enhances ER stress loading in MM cells (PMID: 23546223).　Also, we reported that targeting the integrated networks of aggresome formation, proteasome, and autophagy potentiates ER stress‑mediated cell death in MM cells (PMID: 25422130).

Treatment of MM was greatly improved with the approval of the proteasome inhibitor BTZ in 2003. However, relapse after BTZ treatment is inevitable, and MM remains incurable. Drug resistance in MM can be caused by two primary mechanisms.

The first drug resistance is stromal cell mediated drug resistance. Many studies have shown that MM cells become resistant to chemotherapeutic agents by directly interacting with BMSC, thereby leading to poor survival of patients with MM.

The second drug resistance is due to acquired genetic mutation.

Mutations in 26S PSMB5 (proteasome subunit beta type 5) have been reported in BTZ-resistant MM cells. This mutation resulting in reduced BTZ binding PSMB5 and thus inability to efficiently inhibit the proteasome (PMID: 30026573).

We think that stromal cell mediated drug resistance is the first major factor in drug resistance of MM. In the present study,　we focused on drug resistance via adhesion between bone marrow stromal cells and myeloma cells.

Furthermore, in contrast to previous studies, we focused on NOXA, which is known to be an important molecule in ER stress-mediated cell death. The importance of NOXA in BTZ plus CAM combination therapy was demonstrated in the experiments with NOXA knockout cell lines. In addition, NOXA is degraded by autophagy as well as proteasomes, blocking autophagy by CAM causes a sustained up-regulation of NOXA in MM cells co-cultured with stromal cells.

Overcoming of stromal cell mediated drug resistance may prevent genetic mutations caused by prolonged chemotherapy in MM cells. Therefore, we think that this study is significantly different from previous studies. Thank you for giving us the opportunity to strengthen our manuscript with your valuable comments and queries. Newly, the significance of this study in drug resistance to MM cells is added in the introduction section.

Introduction:

Recently, proteasome subunit beta type 5 (PSMB5) mutations were identified as one of the causes for drug resistance in MM cells chronically exposed to BTZ [14]. That means cell adhesion-mediated drug resistance prolongs the period of BTZ treatment and contributes to the acquisition of drug resistance due to the genetic mutations in the MM cells. Therefore, overcoming of stromal cell mediated drug resistance is important to prevent genetic drug resistance caused by chronic chemotherapy.

We wish to thank the Reviewer again for valuable comments.
